# Effects of Physical Exercise on Sexual Function and Quality of Sexual Life Related to Menopausal Symptoms in Peri- and Postmenopausal Women: A Systematic Review

**DOI:** 10.3390/ijerph17082680

**Published:** 2020-04-14

**Authors:** María del Carmen Carcelén-Fraile, Agustín Aibar-Almazán, Antonio Martínez-Amat, David Cruz-Díaz, Esther Díaz-Mohedo, María Teresa Redecillas-Peiró, Fidel Hita-Contreras

**Affiliations:** 1 Department of Health Sciences, Faculty of Health Sciences, University of Jaén, 23071 Jaén, Spain; mccf0004@red.ujaen.es (M.d.C.C.-F.); amamat@ujaen.es (A.M.-A.); dcruz@ujaen.es (D.C.-D.); fhita@ujaen.es (F.H.-C.); 2 Department of Physiotherapy, University of Málaga, 29016 Málaga, Spain; estherdiaz@uma.es; 3 Hospital San Agustín de Linares, 23700 Linares (Jaén), Spain; mayteredecillas@gmail.com

**Keywords:** sexual function, physical exercise, quality of life, menopause, systematic review

## Abstract

During the menopausal period, sexual dysfunction is associated with the development or worsening of psychological conditions, causing deterioration in women’s mental health and quality of life. This systematic review aims to investigate the effects of different exercise programs on sexual function and quality of sexual life related to menopausal symptoms. With this purpose, a systematic literature search was conducted in PubMed, CINAHL, Scopus, Web of Science, and Cochrane Plus. A total of 1787 articles were identified in the initial search and 11 prospective studies (including 8 randomized controlled trials) were finally included. The most commonly recommended training programs are based on exercising pelvic floor muscles, as they seem to have the largest impact on sexual function. Mind–body disciplines also helped in managing menopausal symptoms. However, as far as the most traditional programs were concerned, aerobic exercises showed inconsistent results and resistance training did not seem to convey any benefits. Although positive effects have been found, evidence supporting physical exercise as a strategy to improve sexual function and quality of sexual life related to menopausal symptoms is limited, and further studies on this topic are needed.

## 1. Introduction

Menopause is a natural period that all women experience with age. It involves a complex interaction of genetic, biological, and psychological factors [1]. The decrease in estrogen levels produces a series of symptoms that impair quality of life (QOL), affecting the physical, mental, and sexual health of women [2]. Regarding the latter, the menopause transition can impair sexual relations by affecting the biological systems involved in normal sexual response [3].

Sexuality is defined as a state of physical, psychological, social, and emotional well-being that is related to sexual desire [4]. Female sexual dysfunction negatively affects QOL and self-esteem, causing emotional distress and relationship problems [5]. Given that women currently spend a third or more of their lives after menopause, it is essential to pay attention to their health problems and sexual difficulties and establish strategies to prevent a reduction of sexual activity in women [6].

A sedentary lifestyle is associated with numerous adverse health outcomes such as cardiovascular disease [7,8] and mortality. In postmenopausal women, physical inactivity has been shown to exacerbate a variety of health problems, including but not limited to those linked to menopause [9]. Physical exercise is one of the most widely recognized non-pharmacological interventions and its benefits on physical and mental health have been reported in older adults [10,11] as well as in postmenopausal women [9,12]. However, little attention has been paid in the literature to the possible links between physical exercise and sexual function. The practice of physical exercise can be a key factor in preventing women’s sexual problems [13], given that many of the physiological mechanisms involved in exercise also play a part in female sexual function [14]. To our knowledge, a few systematic reviews studying the effects of physical exercise on female sexual function have been published, but only pelvic floor muscle (PFM) exercises were analyzed, and they were not conducted in postmenopausal women [15,16,17]. The purpose of this systematic review is to provide an analysis of published data concerning the effects of physical exercise on the sexual function of peri- and postmenopausal women, and to analyze the degree to which quality of sexual life is related to menopausal symptoms.

## 2. Materials and Methods 

The bibliographic search, data extraction, and systematic review were carried out following the PRISMA 2009 guidelines [18].

### 2.1. Eligibility Criteria 

Prospective studies that investigate the effects of a physical exercise intervention (at least one group of the study should have carried out a physical exercise intervention program) on sexual function, and the quality of sexual life related to menopausal symptoms in peri- and postmenopausal women were eligible. The search was limited to English language publications and to studies with human participants.

### 2.2. Information Sources and Search Strategy

A systematic literature search was conducted in the PubMed, CINAHL, Scopus, Web of Science, and Cochrane Plus databases without limiting the publication date. We searched (in the title and abstract fields) for the free terms “sexuality” OR “sexual function” OR “sexual activity” OR “sexual health” AND “physical exercise” OR “exercise” OR “training” OR “physical activity” AND “menopaus *” OR “perimenopaus *” OR “postmenopaus *”. An iterative process was used to ensure that all relevant articles were obtained. The search was conducted from January 10, 2020 to March 10, 2020.

### 2.3. Study Selection and Data Extraction

First, duplicate articles and those for which a summary was not available were discarded. Titles and abstracts were screened to exclude items that did not meet the eligibility criteria described above. Finally, full-text articles were examined to verify whether they met the inclusion criteria. A selection was carried out independently by two of the authors (A.A.A., M.C.F.). Discrepancies were solved by consensus with a third author (F.H.C.). Data extracted included: authors; year of publication; location; population (sample size, age, and distribution of groups); study design, outcomes, and measuring tools of the studies; description of intervention procedures; measurement time points; dropout rates; adverse effects; and main findings. 

### 2.4. Outcomes

The primary outcomes of this study were sexual function and quality of sexual life related to menopausal symptoms. Secondary outcomes included the impact of menopausal symptoms on QOL, general and condition-specific health-related QOL, and symptoms of depression and anxiety.

### 2.5. Study Quality

Two independent authors (A.M.A., E.D.M.) assessed the risk of bias of the studies selected by using the Cochrane Collaboration Risk-of-Bias tool [19]. It consists of seven items classified as either low risk, uncertain risk (when no specific details or description was reported), or high risk (not meeting the criteria). Any disagreements were resolved by consensus or by consulting with a third author (M.R.P.). 

## 3. Results

### 3.1. Inclusion of Studies

**Figure 1:**
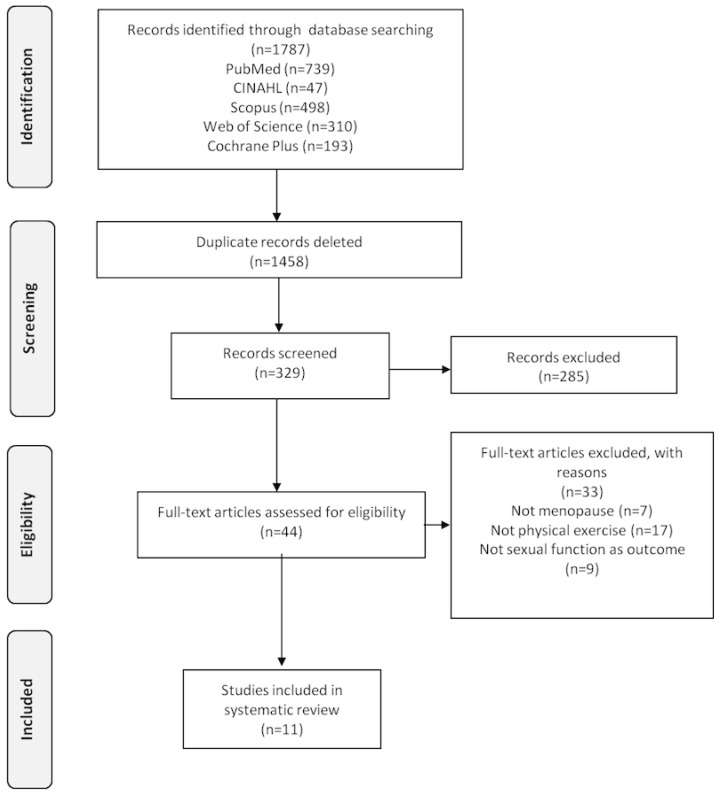
PRISMA flowchart showing the inclusion and exclusion of studies in this systematic review.

Out of the 1787 articles identified in the initial search, 11 publications were finally included in this systematic review. The flowchart of the study selection based on the PRISMA [18] statement is presented in Figure 1.

### 3.2. Quality of Studies

The risk of bias assessment is shown in Table 1. Out of the eight randomized controlled trials (RCTs) included in this systematic review [20,21,22,23,24,25,26,27], only six articles [20,21,22,23,24,25] described the exclusions and losses to follow-up.

### 3.3. Characteristics of Studies and Participants

The findings and full descriptive details of the studies included in this review are presented in Table A1 (see Appendix A). With a total number of eleven articles under analysis, five out of the eight RCTs were two-armed trials [20,22,23,26], two were three-armed [21,24] and one had four arms [27]. Four RCTs were conducted in Asia (two in Iran, one in China, and one in Thailand) [20,21,22,23], four in America (two in the USA, two in Brazil, and one in Canada) [24,25,28,29,30] and two in Europe (both in the Netherlands) [25,27]. A total of 1548 women were selected for participation in the 11 studies included in this systematic review. Mean age, broken down into groups, is displayed in Table A1 (see Appendix A). All studies involved healthy women except four cases in which subjects suffered from primary breast cancer and treatment-induced menopause [27]; genitourinary syndrome of menopause and stress or mixed urinary incontinence [30]; pelvic organ prolapse [26]; and dyspareunia [25]. Pelvic floor muscle (PFM) exercises, either alone [20,21,25] or combined with resistance exercises [28] or physiotherapy treatment [25,30] were the most widely used type of activity. Other studies employed aerobic exercises alone [23,24] or together with resistance exercises [29] or cognitive-behavioral treatment including relaxation exercises. The latter were also employed as an individual intervention [27]. Two articles involved mind–body interventions such as yoga [24] and Rusie Dutton [22], and one performed on women with pelvic organ prolapse employed a silicone pessary [25]. The duration of the interventions was 12 weeks except for those authored by Ngowsiri et al. [22] (13 weeks), Mastrangelo et al. [29] (8 weeks), Panman et al. [26], whose studies on women with pelvic organ prolapse reached 24 months, and Schvartzman et al. [25], who did not describe the exact duration of the intervention (simply reporting five one-hour sessions). The dropout rate was 20.99% (325/1548 participants). Only one of the articles reported adverse effects [26] regarding pelvic floor muscle training (PFMT), while the rest did not provide any statement regarding adverse effects.

### 3.4. Outcomes

Sexual function was assessed by: the Female Sexual Function Index (FSFI), the Sexual Quotient-Female Test, the Sexual Activity Questionnaire, and a binary question (yes/no) regarding improvement in sexual function. In women with pelvic organ prolapse, the Pelvic Organ Prolapse/Incontinence Sexual Function Questionnaire-12 was used. In addition, the International Consultation on Incontinence Questionnaire-Female Lower Urinary Tract Symptoms sex, the International Consultation on Incontinence Questionnaire Vaginal Symptoms (ICIQ-VS) sexual matters subscale, and the Atrophy Symptom Questionnaire (ASQ) sexual function index were employed in women with genitourinary syndrome of menopause and stress or mixed urinary incontinence. Quality of sexual life related to menopausal symptoms was evaluated through the sexual domain of the Menopause-Specific Quality of Life Questionnaire (MENQOL), one item of the modified Kupperman Index (KI), the Hot Flush Rating Scale (HFRS), and the sexuality subscale of the Cervantes questionnaire.

To evaluate the impact of menopausal symptoms on QOL, the following questionnaires were used: KI, MENQOL, HFRS, and the Hot Flash-Related Daily Interference Scale. For generic QOL, The Medical Outcomes Study Short Form Health Survey-12 (SF-12) and -36 (SF-36) were administered, while condition-specific QOL was assessed by the ASQ, the ICIQ-VS for genitourinary syndrome of menopause and stress or mixed urinary incontinence, and the Pelvic Floor Impact Questionnaire-7 for women with pelvic floor prolapse. Finally, anxiety and depression were evaluated by the Hospital Anxiety and Depression Scale.

Concerning the results of the primary outcomes of the present review, the articles under analysis reported a variety of different conclusions. Regarding sexual function, PFM exercises showed significant improvements compared to a control group in some domains of the FSFI such as arousal and orgasm [21], as well as satisfaction. An improvement in sexual function was also reported. In women with genitourinary syndrome of menopause, there were significant improvements in several sexual function indicators after the combination of PFM physiotherapy treatment and home-based PFM exercises [30]. However, no significant improvement in sexual function was observed after resistance exercises combined with PFMT in healthy women [28] and in women with symptomatic pelvic organ prolapse. Additionally, no improvements were observed after PFMT alone, and compared with silicon pessary treatment a significant difference was reported in favor of the latter [26]. Finally, Schvartzman et al. [25] reported that five sessions of PFMT combined with thermotherapy for the relaxation of pelvic floor muscles and myofascial release of PMF trigger points were effective in improving both sexual function and quality of sexual life related to menopausal symptoms.

As for aerobic exercises, Zhang et al. [23] reported improvements in quality of sexual life related to menopausal symptoms after aerobic activity compared to a control group that carried out their activities as usual, but Mastrangelo et al. [29] could not find significant benefits after aerobic and resistance exercises. Yet another study showed improvements after a yoga intervention but not after moderate-intensity aerobic exercises (both with omega-3 supplements) [24]. That same study failed to find significant results regarding sexual function, but a different study involving women with primary breast cancer reported significant benefits in sexual function after 12 weeks of an individually tailored, home-based, aerobic exercise program and after a cognitive-behavioral treatment with relaxation exercises. However, after six months only the latter group retained their improvements [27]. Finally, regarding the primary outcomes of this review, Ngowsiri et al. [22] found improvements in the sexual domain of the MENQOL after 13 weeks of Rusie Dutton exercises, a traditional mind–body exercise which originated in Thailand.

With regard to the secondary outcomes, six articles studied the impact of menopausal symptoms on QOL [22,23,24,25,27,29]. Benefits were reported after an aerobic exercise program, which were significant in all the items of the modified KI and its total score [23], while another two studies showed better scores in the physical domain of the MENQOL [24,29]. The practice of Rusie Dutton showed benefits in the MENQOL physical, psychosocial, and vasomotor domains as well as in the total score [24]. Similarly, yoga seemed to improve the MENQOL vasomotor domain and total score compared to usual activity [22]. Significant increases in the total score and the women and health subscale of the Cervantes questionnaire were reported after PFMT combined with thermotherapy and myofascial release [25]. As for hot flushes and night sweats, results were inconsistent, as improvements (vs. usual activity) could be observed after a yoga intervention [24] and after cognitive-behavioral treatment with relaxation exercises combined with aerobic exercises [27] in women with breast cancer. However, no significant results were observed after an aerobic physical exercise program [23]. Three articles analyzed depression and anxiety, although only PFMT combined with resistance exercises [28] seemed to decrease anxiety symptoms, whereas neither aerobic exercises alone or together with cognitive-behavioral treatment with relaxation exercises produced any significant results [23,27]. No effects were reported in any of these three articles regarding depression. 

Lastly, with respect to generic and condition-specific QOL, better scores in the physical domain of the SF-12 were described in PFMT vs. pessary treatment, in a study involving women with symptomatic pelvic organ prolapse [26]. Duijts et al. [27] used the SF-36 to asses generic QOL, and reported significant improvements in the physical functioning and mental health subscales, as well as in the mental health component score after cognitive-behavioral treatment with relaxation exercises. Additionally, they reported improvements in the vitality and role-emotional subscales when aerobic exercises were added to the previously mentioned treatment. Some of these benefits (role-emotional, mental health, and mental health component score) were maintained after six months, but only by subjects who underwent the combined strategy intervention. Lastly, Mercier et al. [30], after a home-based PFM exercise program combined with a PFM physiotherapy treatment, reported improvements among women with genitourinary syndrome of menopause in the QOL subscale of the ICIQ-VS, as well as in the ASQ total score and in the items that recorded the impact of vaginal dryness and vulvo-vaginal irritation on activities of daily living.

## 4. Discussion

The objective of the present systematic review was to determine the effects of physical exercise on sexual function in postmenopausal women and on menopause-related quality of sexual life. The results of the studies under analysis display a wide range of conclusions. PMF exercises were the most widely used (six articles), with significant improvements in sexual function being reported by four studies (including one that involved women with genitourinary syndrome and another carried out on women with dyspareunia). Concerning the quality of sexual life related to menopausal symptoms, mind–body exercises such as yoga or Rusie Dutton were reported to induce improvements, whereas aerobic and resistance exercise training showed contradictory results. 

The loss of sexual desire related to menopause is a symptom with wide-ranging effects on all aspects of QOL. It has been shown that postmenopause is associated with altered perception of physical appearance and femininity, as well as with mood disturbances, which can in turn influence sexual function [31]. Menopause-related hormonal changes (especially the decrease in estrogen), together with aging, seem to be related to higher risk of sexual dysfunction [32,33]. In addition, vaginal innervations appear to increase as estrogen decreases [34], which may further condition sexual function. It has also been published that common cardiometabolic alterations could affect vascular function in the female genital tract, and that an association exists between cardiovascular risk factors and female sexual health in women [35], although it is less conspicuous than for men. 

A sedentary lifestyle is well known to be associated with adverse physical health outcomes, but also with worsened psychological health [36] including diminished sexual activity and greater sexual problems. Conversely, promoting physical activity among older adults can improve sexual activity [13]. In our review, PFM training was the most commonly used set of exercises (in six studies, five of which were RCTs). In this regard, several systematic reviews have looked into the effects of such training programs on other populations, such as women during their pregnancy and postpartum. Hadizadeh-Talasaz et al. [17], in a recent meta-analysis of clinical trials, reported improvements in sexual function and quality of life in postpartum, but also suggested that high-quality RCTs were needed regarding this topic. Furthermore, Sobhgol et al. [15] concluded that despite the improvements in sexual function brought about by postnatal PFMT alone, there were a lack of studies on pregnant women and available data were limited regarding the postpartum. The domains of sexual function in which improvements were reported were sexual satisfaction, desire, arousal, and orgasm. In yet another systematic review carried out in 2015, women with pelvic floor dysfunction were shown to benefit from pelvic floor exercises, whether alone or combined with other exercises or therapies. Improvements were reported in at least one of the variables under study in relation to sexual function [16]. Both of these systematic reviews concluded that caution must be exercised in the interpretation of their results given the methodological limitations of studies dealing with this matter. The results of the studies included in our review indicate that four out of six interventions involving PFMT reported improvements in sexual function, two of those with PFM exercises alone [20,21] and involving healthy subjects, and the other two in combination with other treatments and performed on a population of women with genitourinary syndrome of menopause [30] and dyspareunia [25].

The literature provides ample evidence of the beneficial effects of physical exercise on hormones, such as oxytocin [37], cortisol [38], or estrogen [39], which seem to affect sexual function and arousal in particular [40]. Similarly, exercise appears to activate the sympathetic nervous system, which is involved in both sexual arousal and orgasm [41]. As for traditional forms of exercise, it has been proven that physical resistance training and aerobic physical training are effective in improving sexual function in women with polycystic ovary syndrome [42,43]. However, among the articles included in our review, aerobic training yielded inconsistent results. For Duijts et al. [27] these exercises did not enhance sexual function, whereas for quality of sexual life related to menopausal symptoms Zhang et al. [23] reported improvements and Reed et al. [24] did not find significant results. Resistance training also failed to induce improvements in the main variables under consideration in our systematic review [28,29]. However, women taking part in a yoga [24] or Rusie Dutton [22] program experienced improvements in their quality of sexual life related to menopausal symptoms, in agreement with reports involving exercise programs based on other mind–body disciplines such as Pilates, which has been shown to be effective in improving sexual function among healthy women [44].

As for the secondary outcomes of this systematic review, the literature includes several publications looking into the effects of a variety of exercise programs on the quality of life of postmenopausal women. For instance, physical training has been described to greatly improve the cardiovascular autonomic nervous system with direct beneficial effects on QOL [45], whereas training exercises have beneficial effects on bone, muscles, and adipose tissue, allowing for increased QOL [46].

As far as menopause-specific QOL is concerned, both aerobic and resistance training, whether alone or together with nutritional supplements or educational initiatives, have been shown to improve climacteric symptoms in postmenopausal women [47,48]. In addition, such exercises alone may positively affect the impact of menopausal symptoms on QOL, as well as on psychological health and depression [49]. In that regard, it has been reported that psychological factors such as anxiety and depression, which are highly prevalent during the female climacteric, negatively affect the sexual life of postmenopausal women [50]. 

Among the findings of the studies included in our review the conclusions were similarly diverse. Concerning resistance training, no significant improvements were described in QoL in general or QoL associated with the symptoms of menopause, with the exception of physical appearance in a program in which resistance was combined with aerobic exercises [29]. On the other hand, Lara et al. [28], in a program combining PFMT and resistance exercises, observed improvements in symptoms of anxiety, but not of depression. In a study dealing with the effects of aerobic exercises, Zhang et al. [23] described benefits in the quality of sexual life related to menopausal symptoms, although other studies failed to find any benefit beyond the physical domains of QoL [24]. Duijts et al. [27] found that the combination of aerobic activity with cognitive-behavioral treatment and relaxation exercises had beneficial effects on QoL in general, but did not observe significant improvements in depression and anxiety symptoms. Nevertheless, these results may reflect on the fact that participants in their study were women with breast cancer. Finally, women who underwent a program including mind–body exercises, whether yoga [24] or Rusie Dutton [22], experienced an improvement in their quality of sexual life related to menopausal symptoms.

This systematic review has some limitations. Most of the studies under analysis here did not go beyond the immediate effects of their interventions. In addition, the exercise programs they employed are highly heterogeneous, as are their methods for assessing sexual function. Future studies, and RCTs in particular, should look into the short-, middle-, and long-term effects of such programs in order to produce a better understanding of the effectiveness of physical exercise in improving sexual function among peri- and postmenopausal women. 

## 5. Conclusions

After a systematic review of studies dealing with the effects of physical exercise programs on sexual function and quality of sexual life related to menopausal symptoms, the results do not allow for clear conclusions. On the one hand, pelvic floor muscles exercises are the most common type of exercise in these studies and the one that seems most beneficial for sexual function, similarly to how mind–body disciplines improve the impact of menopausal symptoms on the quality of sexual life. However, concerning the most traditional forms of exercise, aerobic training yielded inconsistent results and resistance training failed to produce any improvement. This disparity in results, together with the high degree of variability among the exercise programs and assessment methods employed, suggest that any conclusion must be drawn with great caution.

## Figures and Tables

**Table 1:** Assessment of risk of bias for the clinical trials included.

Articles	Random Sequence Generation (Selection Bias)	Allocation Concealment (Selection Bias)	Blinding of Participants and Personnel (Performance Bias)	Blinding of Outcome Assessment (Detection Bias)	Incomplete Outcome Data (Attrition Bias)	Selective Reporting (Reporting Bias)	Other Bias
Schvartzman et al. [25]	L	L	H	H	L	L	L
Nazarpour et al. [20]	L	U	H	U	L	H	L
Nazarpour et al. [21]	L	U	H	U	L	H	L
Panman et al. [26]	L	L	H	L	L	L	L
Zhang et al. [23]	U	U	H	U	H	L	L
Reed et al. [24]	L	U	H	U	U	L	L
Ngowsiri et al. [22]	U	U	H	U	L	L	L
Duijts et al. [27]	L	U	H	U	L	L	L

L: Low Risk. H: High Risk. U: Unclear.

## Appendix A

**Table A1:** Characteristics of the included studies (n = 11).

Study, Year and Location	Studied Population, Groups and Study Design	Outcomes and Measuring Tools	Intervention	Measure Time Points, Dropout and Adverse Effects	Main Findings
Mercier et al. [30] Montréal, Canada.	32 postmenopausal women (≥ 55 years, mean age of 68.0 ± 6.6) with genitourinary syndrome of menopause and stress or mixed urinary incontinence. Design: A single-arm feasibility study embedded in a randomized controlled trial	Primary outcome: Sexual function (ICIQ-FLUTSsex, sexual function item of the ASQ and sexual matters subscale of the ICIQ-VS) Secondary outcomes: Condition-specific QoL (total score and 4 items of the ASQ, QoL subscale of the ICIQ-VS)	Exercise intervention (12 weeks): -Sessions of 1 hour: An intensive PFM physiotherapy treatment with a 15-minute educational part and a 45-minute exercise part (strength, resistance, PFM coordination, and functional PFMT exercises) -5 days per week: four home-based PFM exercises (9-30 repetitions).	Measurements: -At baseline. -At 12 weeks. Dropout: 3 (9.06%) Adverse effects: No adverse effects were reported.	Within-group comparisons: -Pre1-post: There were improvements in dyspareunia impact on sexual function (ASQ), sexual matters subscale of the ICIQ-VS, and ICIQ-FLUTSsex score (*p* = 0.004, *p* = 0.001 and *p* = 0.014 respectively). Benefits were also observed in QoL subscale of the ICIQ-VS (*p* < 0.001) and in ASQ, more precisely in the total score (*p* < 0.001) as well as in the items vaginal dryness (*p* = 0.001) and vulvo-vaginal irritation (*p* = 0.004) impact on activities of daily living. -Pre2-post: Improvements were described in ASQ items dyspareunia impact on sexual function (*p* = 0.004), and in the ASQ total score (*p* < 0.001), as well as in the ASQ items vaginal dryness (*p* = 0.041) and vulvo-vaginal irritation (*p* = 0.001) impact on activities of daily living.
Schvartzman et al. [25] Porto Alegre, Brazil	42 peri- and postmenopausal women aged 40-60 years with dyspareunia (at least 6 months). EXP 1 (n = 21, 51.9 ± 5.3 years) EXP 2 (n = 21, 50.6 ± 4.7 years) Design: Randomized controlled trial	Primary outcome: Sexual function (FSFI). Quality of sexual life related to menopausal symptoms (Cervantes Scale sexuality subscale). Secondary outcome: Impact of menopausal symptoms on QoL (Cervantes Scale).	-EXP1: 5 sessions (1 hour) of thermotherapy for relaxation of pelvic floor muscles, myofascial release of PMF trigger points, and PFMT. -EXP2: 5 sessions (1 hour), heat applied to the lower back with myofascial release of abdominal diaphragm, piriformis, and iliopsoas muscles. No PFMT.	Measurements: -At baseline -After the intervention. Dropout: EXP1: 1 (4.76%) EXP2: 3 (14.86%) Adverse effects: Not mentioned.	Within-group comparisons: EXP1: improvements in all FSFI subscales and total score, as well as in menopause and health and sexuality Cervantes subscales and total score. Between-group comparisons: EXP1: improvements in lubrication and pain FSFI subscales and total score, as well as in menopause and health Cervantes subscale.
Nazarpour et al. [20] Chalous and Noshahr, Iran.	104 postmenopausal women (40-60 years): -EXP (n = 52, 53.3 ± 2.67 years). -CON (n = 52, 52.84 ± 3.99 years). Design: Randomized controlled trial.	Primary outcome: Sexual function (FSFI). Improvement in sexual function (binary question).	-EXP: 12 weeks. PFM exercises (contraction of the muscles for 10s, relaxation for 10 s and repetition for 10 times in 3-4 sessions a day) with written material, images and videos. -CON: received general information as to the physiological and psychological symptoms of menopause. Both groups also received sex educational booklets.	Measurements: -At baseline. -At 12 weeks. Dropout: EXP: 5 (9.62%) CON: 2 (3.84%) Adverse effects: Not mentioned.	Between-group comparisons: Compared to CON, EXP showed significantly higher scores only in FSFI domains arousal (*p* = 0.034), orgasm (*p* = 0.028) and satisfaction (*p* = 0.011), and reported more improvement in their sexual function (binary question, *p* = 0.004).
Nazarpour et al. [21] Chalous and Noshahr. Northerm Iran.	156 postmenopausal women (40-60 years): -EXP1 (n = 52, 51.5 ± 3.4 years). -EXP2 (n = 52, 53.1 ± 2.7 years). -CON (n = 52, 52.9 ± 4.0 years). Design: Randomized controlled trial.	Primary outcome: Sexual function (FSFI). Improvement in sexual function (binary question).	-EXP1: 12 weeks. Formal sex education program focused on various aspects of sexual function during menopause. EXP2: 12 weeks. PFMT (Kegel exercises using oral descriptions, written material, images, and videos, and checklists for daily exercise). -CON: participants received general educational material about menopause.	Measurements: -At baseline. -At 12 weeks. Dropout: EXP1: 4 (7.69%) EXP2: 5(9.62%) CON: 2(3.85%) Adverse effects: Not mentioned.	Between-group comparisons: Compared to CON, both EXP1 and EXP2 showed significantly higher scores in arousal FSFI domain (*p* < 0.05), and improvement in sexual function (binary question, *p* < 0.001), while only EXP2 showed significantly better scores in orgasm (*p* = 0.015).
Panman et al. [26] Groningen, the Netherlands.	162 women aged ≥ 55 years with pelvic organ prolapsed: -EXP1 (n = 80 and 4 did not received treatment as randomized. 65.6 ± 6.4 years.) -EXP2 (n = 82 and 35 did not received treatment as randomized. 64.9 ± 7.4 years.) Design: Randomized controlled trial.	Primary outcome: Sexual function (PISQ-12). Secondary outcome: Condition-specific QoL (PFIQ-7) Generic QoL (physical and mental components of the SF-12).	-EXP1: 3-5 times per week, 2-3 times each day. PFMT with specific exercises adapted to the needs of each participant. -EXP2: Silicone pessary treatment.	Measurements: -At baseline. -At 24 months Dropout: EXP1: 9 (11.25%) and 10 (12.50%) discontinued training protocol. EXP2: 8 (9.76%) and 12 (14.63%) discontinued pessary treatment Adverse effects: EXP1: increased vaginal discharge (n = 14) and urinary incontinence (n = 5), irritation or erosions of the vaginal walls on physical examination (n = 10). EXP2: No adverse effects were reported.	Between-group comparisons: 1. Intention to treat analysis (all participants): No significant differences were observed. 2. Per-protocol analysis (participants who completed the intervention to which they were allocated): EXP2 showed significant improvements in sexual function (*p* = 0.028), and EXP1 in the physical component of the SF-12 (*p* = 0.004). No other significant differences were reported.
Ngowsiri et al. [22] Bangkok, Thailand.	54 postmenopausal women (45-59 years): -EXP (n = 27, 52.9 ± 4.3 years). -CON (n = 27, 50.7 ± 3.6 years). Design: Randomized controlled trial.	Primary outcome: Quality of sexual life related to menopausal symptoms (MENQOL sexual domain). Secondary outcomes: Impact of menopausal symptoms on QoL (MENQOL vasomotor, physical and psychosocial domains).	-EXP: 13 weeks. Week 1: One session (120 minutes) which consisted of Rusie Dutton exercise explanation and training in deep breathing techniques. Weeks 2-13: Rusie Dutton practice sessions (90 minutes) performed 3 times in the second week, twice in class and once at home in the third week, once a week in class and at least 2 days/week. -CON: No intervention. Both groups received a menopausal health promotion manual.	Measurements: -At baseline. -At 13 weeks. Dropout: EXP: 3 (12.5%) CON: 1 (3.85%) Adverse effects: Not mentioned.	Within-group comparisons: EXP improvements in MENQOL-sexual domain (*p* = 0.004), MENQOL-vasomotor domain (*p* = 0.005), MENQOL-psychosocial domain (*p* = 0.010) and MENQOL-physical domain (*p* = 0.002). Between-group comparisons: Compared to CON, EXP showed significant improvements in MENQOL-sexual domain (*p* = 0.003), as well as in MENQOL-vasomotor domain (*p* = 0.040), MENQOL-psychosocial domain (*p* = 0.000) and MENQOL-physical domain (*p* = 0.000).
Reed et al. [24] Indianapolis, Oakland, and Seattle, USA.	355 peri and postmenopausal women (40-55 years): -EXP1 (n = 107, 54.3 ± 3.9 years). -EXP2 (n = 106, 55.8 ± 3.6 years). -CON (n = 142, 54.2 ± 3.5 years). Design: Randomized controlled trial.	Primary outcomes: Sexual function (FSFI), Quality of sexual life related to menopausal symptoms (MENQOL sexual domain) Secondary outcomes: Impact of menopausal symptoms on QoL (MENQOL, HFRDIS).	-EXP1: 12 weeks. Once per week. Sessions of 90 minutes. Yoga (breathing exercises, 11-13 poses and guided meditation). Daily home (20 minutes) on days not attending class. -EXP2: 12 weeks. 3 times per week. Sessions of 40-60 minutes. Moderate-intensity aerobic exercise. Individual cardiovascular conditioning training sessions at 50-60% of the heart rate reserve (first month) and 60-70% (the remainder of the intervention). -CON: usual physical activity. Before allocation to EXP1, EXP2 and CON, all women received either a placebo containing olive oil or an active omega-3 capsule.	Measurements: -At baseline. -At 12 weeks. Dropout: EXP1: 22 (20.56%) EXP2: 27 (25.47%) CON: 25 (17.61%) Adverse effects: Not mentioned.	Between-group comparisons: Compared to CON: EXP1 showed improvements after intervention in MENQOL sexual domain (*p* = 0.03) as well as in MENQOL total score (*p* = 0.02), MENQOL vasomotor domain (*p* = 0.02), and hot flash-related daily interference assessed with the HFRDIS (*p* = 0.03). Compared to CON, EXP2 showed improvements after intervention in MENQOL physical domain (*p* = 0.02). No other significant results were observed.
Zhang et al. [23] Beijing, China.	157 perimenopausal women (40 to 55 years, KMI ≥ 15): -EXP (n = 78, 47.82 ± 4.58 years). -CON (n = 79, 48.64 ± 5.24 years). Design: Randomized controlled trial.	Primary outcome: Quality of sexual life related to menopausal symptoms (one item of the modified KI). Secondary outcomes: Impact of menopausal symptoms on QoL (Modified KI score).	-EXP: 12 weeks. 3 times per week. Sessions of 30 minutes. The aerobic physical activity was walking with strides of 60-70 cm long, completing 100 m within 60-70 seconds. In addition, this group attended a collective exercise sessions. CON: usual activity	Measurements: -At baseline. - At 4 weeks. -At 8 weeks. -At 12 weeks. Dropout: EXP: 24 (30.77%) CON: 22 (27.85%) Adverse effects: Not mentioned.	Within-group comparisons: -EXP showed improvements in sexual life (one item of the modified KMI, (*p* < 0.005) as well as in total modified KMI total score (*p* < 0.001) and all KMI items. Between-group comparisons: -After intervention, and compared to CON, EXP showed improvements in sexual life (*p* < 0.05) and modified KMI total score (*p* < 0.001), as well as other modified KMI items: paresthesia (*p* < 0.05), insomnia (*p* < 0.05), irritability (*p* < 0.001), fatigue (*p* < 0.05), bone/joint/muscle pain (*p* < 0.001), headache (*p* < 0.05), formication (*p* < 0.05).
Duijts et al. [27] Amsterdam and Rotterdam, the Netherlands.	422 women with primary breast cancer (stages, T1-4, N0-1, and M0), with treatment-induced menopause (48.2 ± 5.6 years). -EXP1 (n = 109, 48.2 ± 5.7 years) -EXP2 (n = 104, 47.7± 5.6 years) -EXP3 (n = 106, 49.0 ± 4.9 years) -CON (n = 103, 47.8 ± 6.0 years) Design: Randomized controlled trial.	Primary outcome: Sexual function (SAQ). Secondary outcomes: Quality of sexual life related to menopausal symptoms (HF/NS problems assessed with HFRS), depressive and anxiety symptoms (HADS), generic QoL (SF-36).	-EXP1: 12 weeks. 6 times per week. Sessions of 90 minutes. Cognitive-behavioural treatment with relaxation exercises. A booster session was held 6weeks after completion of the program. -EXP2: 12 weeks. 2.5-3 hours per week. Individually tailored, self-directed exercise program (swimming, running, cycling, etc.). Target heart rate: 60% to 80% Karvonen. Last week: final session in which they received advice on the best way to maintain the desired level of physical activity -EXP3: underwent EXP1 and EXP2 simultaneously. -CON: No intervention.	Measurements: -At baseline. -At 12 weeks. -At 6 months. Dropout: 1. All patients: -At 12 weeks: EXP1: 23 (21.10%) EXP2: 17 (16.35%) EXP3: 16 (15.09%) CON: 14 (13.59%) -At 6 months: EXP1: 21 (19.27%) EXP2: 25 (24.04%) EXP3:17 (16.04%) CON: 19 (18.45%) 2. Patients who did not meet criteria for minimal compliance with the intervention(s). EXP1: 58% EXP2: 64% EXP3: 70% Adverse effects: Not mentioned.	Between-group comparisons (compared to CON): 1. Intention to treat analysis (all participants): -At 12 weeks, EXP1 (*p* = 0.001), EXP2 (*p* < 0.001) and EXP3 (*p* = 0.001) showed improvements at SF-36 physical functioning domain. -At 6 months, EXP1 (*p* = 0.042) and EXP3 (*p* = 0.002) exhibited significant improvements in sexual function (SAQ habit subscale), while only EXP2 (*p* = 0.002) showed improvements regarding SF-36 physical functioning subscale 2. Per-protocol analysis (participants who met criteria for compliance). Only EXP1 and EXP 3 showed improvements: -At 12 weeks, significant benefits were described for EXP1 at SAQ- pleasure subscale (*p* = 0.002), HF/NS problem (*p* < 0.001), and SF-36 subscales of physical functioning (*p* = 0.003), vitality (*p* = 0.002), mental health (*p* = 0.042) and mental component (*p* = 0.015). For EXP 3, improvements were observed in HF/NS problem (*p* < 0.001), and SF-36 subscales of physical functioning (*p* = 0.003), vitality (*p* = 0.009), role emotional (*p* = 0.001), mental health (*p* = 0.002) and mental component (*p* = 0.002). -At 6 months, improvements were found for EXP1only at SAQ- pleasure subscale (*p* = 0.022) and HF/NS problem (*p* < 0.001), while EXP3 showed benefits in HF/NS problem (*p* < 0.001), and SF-36 subscales of role emotional (*p* = 0.033), mental health (*p* = 0.002) and mental component (*p* = 0.019).
Lara et al. [28] São Paulo, Brasil.	45 sedentary postmenopausal women (46–58 years, mean age of 52.1 ± 3.5 years, not > 5 years of menopause). Design: Prospective, longitudinal exploratory study.	Primary outcome: Sexual function (SQ-F). Secondary outcomes: Anxiety and depression (HADS).	Exercise intervention: 12 weeks. 2 times per week. Sessions of 60 minutes. Physical exercise program which consists of 4 sets of 10 repetitions of maximal PFMT. The program also included warm-up exercises (10 minutes), stretching (10 minutes), muscle strengthening exercises (35 minutes), and relaxation (5 minutes). In addition, they had to do exercises at home three times a week.	Measurements: -At baseline. -At 12 weeks. Dropout: 13 (28.88%) Adverse effects: Not mentioned.	Within-group comparisons: After exercise intervention, there were no significant results regarding sexual function. The number of women with anxiety was significantly less (*p* < 0.01), but any other results were found.
Mastrangelo et al. [29] New Jersey, USA.	19 peri- and postmenopausal women aged 46-63 years old (mean age 54.8 years). Design: Pre- to post-test of convenience.	Primary outcomes: Quality of sexual life related to menopausal symptoms (MENQOL-sexual domains). Secondary outcomes: Impact of menopausal symptoms on QoL (MENQOL vasomotor, physical and psychosocial domains).	Exercise intervention: 8 weeks. 2 times per week. Sessions of 50 minutes. The training consisted of a 5-minute cardiovascular warm-up (on a stationary bicycle), a 40-minute circuit training program (aerobic and resistance exercises, with 10 hydraulic machines and 3 types of aerobic exercise), and finished with a 5-minute stretch/cool down.	Measurements: -At baseline. -At 8 weeks. Dropout: 7(36.84%). Adverse effects: Not mentioned.	Within-group comparisons: After exercise intervention, there were no significant results regarding sexual function or any other domains of the MENQOL, except for the physical domain (*p* = 0.008).

ASQ, Atrophy symptom questionnaire; CON, Control group; EXP, Experimental group; FSFI, Female Sexual Index; HFRDIS, Hot Flash-Related Daily Interference Scale; HADS, Hospital Anxiety and Depression Scale; HF/NS, hot flashes and night sweats; HFRS, Hot Flush Rating Scale; ICIQ-FLUTSsex, International Consultation on Incontinence Questionnaire- Female Lower Urinary Tract Symptoms sex; ICIQ-VS, International Consultation on Incontinence Questionnaire Vaginal Symptoms; KI, Kupperman Index; MENQOL, Menopause-Specific Quality of Life Questionnaire; PFIQ-7, Pelvic Floor Impact Questionnaire-7; PFM, Pelvic floor muscle; PFMT, pelvic floor muscle training; PISQ-12, Pelvic Organ Prolapse/Incontinence Sexual Function Questionnaire-12; QoL, Quality of Life; SF-12, Medical Outcomes Study Short Form Health Survey-12; SF-36, Medical Outcomes Study Short Form Health Survey-36; SQ-F, Sexual Quotient-Female Test; SAQ, Sexual Activity Questionnaire.
